# Supplementary material for: Inverse-Folding Design of Yeast Telomerase RNA Increases Activity In Vitro
Source: Noncoding RNA. 2023 Aug 28;9(5):51. doi: 10.3390/ncrna9050051 (PMC10514824; doi:10.3390/ncrna9050051)
Supplement: Supplementary file 1 [file ncrna-09-00051-s001.zip › ncrna-2548901-supplementary.pdf]

Supplemental Figure S1

RNA nucleotide sequences of inverse-designed TLC1 alleles

Nucleotide substitution mutations were made in TLC1, without deletions or insertions, in non-essential regions previously deleted in Mini-T (Zappulla *et al.*, *NSMB* 2005) or stiffened in TSA-T (Lebo and Zappulla, *RNA* 2012). Mutated nucleotides are indicated in red. The terminal arm is highlighted in green (with 15 nucleotide substitutions), the Ku arm in orange (with 11–62 substitutions), the Est1 arm in blue (with 33 substitutions), and the template in gray.

(A) TLC1

(B) DA-TLC1

(C) DET-TLC1

(D) DK-TLC1

(E) DPhyK-DA-TLC1

(F) DPhyK-TLC1

Supp. Fig. S1A: TLC1

1	GAGAGGAAGAUAGGUACCCUAUGAAAAUGUCAAUGGCUGUUGCGUUUGCU	Terminal arm (5')
51	UAAUCGUAUUUUUUUUUUUUCAGUCCGUGUUUUUGUACAUUCUACGUU	
101	UGAGUUUUCCAUCAUGCAGGCCUCAGAAAUUUGGUAGGCACUCGAUGGUG	
151	AAGAGAUAGUGUCGGAUUUCGGAUUGAUCUUUCAGUUGAUAGCCUGCUGC	
201	UCUUUUCUUUUCCAAAGAAUUUCGAGUAUGCUGGUGUCAGUGUAGAUGCU	Ku arm
251	UGUGUGUGCGCAAUUUGUGGUUUUUUAUUGUGUUUCUACUUAUAGAUGGC	
301	UAAAAUCUGAGUUUAGAAAAUGCAAACCGUAAAUUCUUAAACACUGCUAU	
351	UGCAUUUAGUUGCUAAAGCAGUGUUUUUGAACUUAUUCCUGUUAUUCCUU	
401	CUUCGUACCGAUCCUCUUCUCGACCUAACCUUUUAAUUACCAUGGGAAGC	
451	CUACCAUCACCACACCCACACACAAAUGUUACAGCUAAUUGUUUAUUAGC	template
501	AAAGUUUGCACGAGUUCGCUGUUUAUUUUUUUCUCGUUUUCUUAUACCUA	
551	GUAUUUUUUCUGACACUGUUUAAGGUGACAGAAAAAAAAGGAGUUUAAGUU	Est1 arm
601	AGAUUUGCAAACAGACGGUGCUAAGCGCUGUCACUUUAUGUCUAUCUUAU	
651	CGUUAACUCUGGAAAAAGAAAAAGGAAAAAGAACGUCAGGGAACAUGAGU	
701	AUAUAUAGAAAUGGUUUAUUCUAGUUUUUUCCGUUUUUUCAGUAGAUUUU	
751	UGCCUUUAAAAGAAUAAAUCCCACUACAAAAAGGUAAAAUAAAAAAUCUA	
801	UUCACUGAACUUACUGAUGAAAUUUCCAAAUGUGCCCCGUACAUCGAACG	
851	AUGUGACAGAGAAAAAUACGAGUAGGUAAAUAAGCCAAAAGGCAAGGGUG	
901	UCCUUUCUUAAGCAUCGGUUAGGUUUGCGGGCGAUCAGUAACUGAACAAU	
951	GACACAAGAUCAAGAACGUAAUUUGAGAUUUUUCAAGAUGGUUUUUUUAG	Terminal arm (3')
1001	GUAUCUAUUAAAACUACUUUGAUGAUCAAUACGGUAUUUUUGUCGCAUUA	
1051	UUUUCCAAGCGGAAGGAACCGUGUGUUCAUUUUAUGAAUCUUGGUGUUGU	
1101	AUUCACAGCUACUUCUCCUAAUGCCUUCGAUGCAUUUAGAUAAUUUUUGG	
1151	AAACAUU	

Supp. Fig. S1B: DA-TLC1

1 GAGAGGAAGAUAGGUACCCUAUGAAAAUGCCAAUGGCUGUUGCGUUUGCC
51 UAAUCGUAUUUUUCUCUUUUUCAGUCCGUGUUCUUUGUACAUUCCACGUU

Terminal arm (5')

101 UGAGUGUGCCAUCAUGCAGGCCUCAGAAAUUUGGUAGGCACCCGAUGGUG
151 AAGAGACAGUGUCGGCUUUCGGACCGAUCUUUCAGUUGAUAGCCUGCUGC
201 UCUUUUCUUUUCCAAAGAAUUUCGAGUAUGCUGGUGUCAGUGUAGAUGCU
251 UGUGUGUGCGCAAUUUGUGGUUUUUUAUUGUGUUUCUACUUAUAGAUGGC
301 UAAAAUCUGAGUUUAGAAAAUGCAAACCGUAAAUUCUUAAACACUGCUAU
351 UGCAUUUAGUUGCUAAAGCAGUGUUUUUGAACUUAUUCCUGUUAUUCCUU
401 CUUCGUACCGGUCCUUUGCUCGACCUGACCUCUUAAUCACCAUGGGAAGC

Ku arm

451 CUACCAUCACCACACCCACACACAAAUGUUACAGCUAAUUGUUUAUUAGC

template

501 AAAGUUUGGUCGACUCCUCUGUUUAUCGUGUUCCAAGUGUCCUUUUCCUA
551 GUAUUUUUUCUGACACUGUUUAAGGUGACAGAAAAAAAGGAGUUUAAGUU
601 AGAUUUGCAAACAGACGGUGCUAAGCGCUGUCACUUUAUGUCUAUCUUAU
651 CGUUAACUCUGGUUUAGGACACAGGAACACGACCUUCAGUGGAGACGUCC

Est1 arm

701 AUAUAUAGAAAUGGUUUAUUCUAGUUUUUUCCGUUUUUUCAGUAGAUUUU
751 UGCCUUUAAAAGAAUAAAUCCCACUACAAAAAGGUAAAAUAAAAAAUCUA
801 UUCACUGAACUUACUGAUGCACUCUCCAAACGUGCCCCGUACAUCGAACG
851 ACGUGACAGAGAAAAAUACGAGUAGGCAAACAAGCCAAAAGGCAAGGGUG
901 UCCUUUCUUAAGCAUCGGUUAGGUUUGCGGGCGAUCAGUAACUGAACAAU
951 GACACAAGAUCAAGAACGUAAUUUGAGAUUUUUCAAGAUGGUUUUUUUAG
1001 GUAUCUAUUAAAACUACUUUGAUGAUCAAUACGGUAUUUUUGUCGCAUUA
1051 UUUUCCAAGCGGAAGGAACCGUGUGUUCAUUUUAUGAAUCUUGGUGUUGU
1101 AUUCACAGCUACUUCUCCUAAUGCCUUCGAUGCAUUUAGAUAAUUUUUGG
1151 AAACAUU

Terminal arm (3')

Supp. Fig. S1C: DET-TLC1

Terminal arm (5')

1 GAGAGGAAGAUAGGUACCCUAUGAAAAUGCCAAUGGCUGUUGCGUUUGCC

51 UAAUCGUAUUUUUCUCUUUUUCAGUCCGUGUUCUUUGUACAUUCCACGUU

101 UGAGUGUGCCAUCAUGCAGGCCUCAGAAAUUUGGUAGGCACUCGAUGGUG

Ku arm

151 AAGAGAUAGUGUCGGAUUUCGGAUUGAUCUUUCAGUUGAUAGCCUGCUGC

201 UCUUUUCUUUUCCAAAGAAUUUCGAGUAUGCUGGUGUCAGUGUAGAUGCU

251 UGUGUGUGCGCAAUUUGUGGUUUUUUAUUGUGUUUCUACUUAUAGAUGGC

301 UAAAAUCUGAGUUUAGAAAAUGCAAACCGUAAAUUCUUAAACACUGCUAU

351 UGCAUUUAGUUGCUAAAGCAGUGUUUUUGAACUUAUUCCUGUUAUUCCUU

401 CUUCGUACCGAUCCUCUUCUCGACCUAACCUUUUAAUUACCAUGGGAAGC

template

451 CUACCAUCACCACACCCACACACAAAUGUUACAGCUAAUUGUUUAUUAGC

Est1 arm

501 AAAGUUUGGUCGACUCCUCUGUUUAUCGUGUUCCAAGUGUCCUUUUCCUA

551 GUAUUUUUUCUGACACUGUUUAAGGUGACAGAAAAAAAGGAGUUUAAGUU

601 AGAUUUGCAAACAGACGGUGCUAAGCGCUGUCACUUUAUGUCUAUCUUAU

651 CGUUAACUCUGGUUUAGGACACAGGAACACGACCUUCAGUGGAGACGUCC

701 AUAUAUAGAAAUGGUUUAUUCUAGUUUUUUCCGUUUUUUCAGUAGAUUUU

751 UGCCUUUAAAAGAAUAAAUCCCACUACAAAAAGGUAAAAUAAAAAAUCUA

Terminal arm (3')

801 UUCACUGAACUUACUGAUGCACUCUCCAAACGUGCCCCGUACAUCGAACG

851 ACGUGACAGAGAAAAAUACGAGUAGGCAAACAAGCCAAAAGGCAAGGGUG

901 UCCUUUCUUAAGCAUCGGUUAGGUUUGCGGGCGAUCAGUAACUGAACAAU

951 GACACAAGAUCAAGAACGUAAUUUGAGAUUUUUCAAGAUGGUUUUUUUAG

1001 GUAUCUAUUAAAACUACUUUGAUGAUCAAUACGGUAUUUUUGUCGCAUUA

1051 UUUUCCAAGCGGAAGGAACCGUGUGUUCAUUUUAUGAAUCUUGGUGUUGU

1101 AUUCACAGCUACUUCUCCUAAUGCCUUCGAUGCAUUUAGAUAAUUUUUGG

1151 AAACAUU

Supp. Fig. S1D: DK-TLC1

1 GAGAGGAAGAUAGGUACCCUAUGAAAAUGUCAAUGGCUGUUGCGUUUGCU
51 UAAUCGUAUUUUUUUUUUUUUCAGUCCGUGUUUUUGUACAUUCUACGUU
101 UGAGUUUUCCAUCAUGCAGGCCUCAGAAAUUUGGUAGGCACCCGAUGGUG

Terminal arm (5')

151 AAGAGACAGUGUCGGCUUUCGGACCGAUCUUUCAGUUGAUAGCCUGCUGC
201 UCUUUUCUUUUCCAAAGAAUUUCGAGUAUGCUGGUGUCAGUGUAGAUGCU
251 UGUGUGUGCGCAAUUUGUGGUUUUUUAUUGUGUUUCUACUUAUAGAUGGC
301 UAAAAUCUGAGUUUAGAAAAUGCAAACCGUAAAUUCUUAAACACUGCUAU
351 UGCAUUUAGUUGCUAAAGCAGUGUUUUUGAACUUAUUCCUGUUAUUCCUU
401 CUUCGUACCGGUCCUUUGCUCGACCUGACCUCUUAAUCACCAUGGGAAGC

Ku arm

451 CUACCAUCACCACACCCACACACAAAUGUUACAGCUAAUUGUUUAUUAGC

template

501 AAAGUUUGCACGAGUUCGCUGUUUAUUUUUUUCUCGUUUUCUUAUACCUA
551 GUAUUUUUUCUGACACUGUUUAAGGUGACAGAAAAAAAGGAGUUUAAGUU
601 AGAUUUGCAAACAGACGGUGCUAAGCGCUGUCACUUUAUGUCUAUCUUAU
651 CGUUAACUCUGGAAAAAGAAAAAGGAAAAAGAACGUCAGGGAACAUGAGU

Est1 arm

701 AUAUAUAGAAAUGGUUUAUUCUAGUUUUUUCCGUUUUUUCAGUAGAUUUU
751 UGCCUUUAAAAGAAUAAAUCCCACUACAAAAAGGUAAAAUAAAAAAUCUA
801 UUCACUGAACUUACUGAUGAAAUUUCCAAAUGUGCCCCGUACAUCGAACG
851 AUGUGACAGAGAAAAAUACGAGUAGGUAAAUAAGCCAAAAGGCAAGGGUG
901 UCCUUUCUUAAGCAUCGGUUAGGUUUGCGGGCGAUCAGUAACUGAACAAU
951 GACACAAGAUCAAGAACGUAAUUUGAGAUUUUUCAAGAUGGUUUUUUUAG
1001 GUAUCUAUUAAAACUACUUUGAUGAUCAAUACGGUAUUUUUGUCGCAUUA
1051 UUUUCCAAGCGGAAGGAACCGUGUGUUCAUUUUAUGAAUCUUGGUGUUGU
1101 AUUCACAGCUACUUCUCCUAAUGCCUUCGAUGCAUUUAGAUAAUUUUUGG
1151 AAACAUU

Terminal arm (3')

Supp. Fig. S1E: DPhyK-DA-TLC1

1 GAGAGGAAGAUAGGUACCCUAUGAAAAUGCCAAUGGCUGUUGCGUUUGCC
51 UAAUCGUAUUUUUCUCUUUUUCAGUCCGUGUUCUUUGUACAUUCCACGUU
101 UGAGUGUGCCAUCAUGCAGGCCUCAGAAAUUUGGUAGGCACCCGAUGGUG
151 AAGAGACAGUGUCGGCUUUCGGACCGUUCUUUUCGGUCAGAGCCUGUCGC
201 UCUUCUCUCUUCCAGAGAGUUUCGUGCACGCUGGCGUCAGUGUAGACGCC
251 UGUGCGUGCUCAAUUUGUGGUUUUUAUUGUGUUUCUACUUAUAGAUGGC
301 UAAAAUCUGAGUUUAGAAAAUGCAAACCGUAAAUUCUUCUUUCUUCUUCU
351 UGCGUUCAGUUGCUCAACCAGUGUUUUUCCGGUUAUUCCUCCGUUUCUUU
401 CUUUCUUUCGGUCCUUUGCUCGACCUGACCUCUUAAUCACCAUGGGAAGC
451 CUACCAUCACCACACCCACACACAAAUGUUACAGCUAAUUGUUUAUUAGC
501 AAAGUUUGGUCGACUCCUCUGUUUAUCGUGUUCCAAGUGUCCUUUUCCUA
551 GUAUUUUUUCUGACACUGUUUAAGGUGACAGAAAAAAAGGAGUUUAAGUU
601 AGAUUUGCAAACAGACGGUGCUAAGCGCUGUCACUUUAUGUCUAUCUUAU
651 CGUUAACUCUGGUUUAGGACACAGGAACACGACCUUCAGUGGAGACGUCC
701 AUAUAUAGAAAUGGUUUAUUCUAGUUUUUUCCGUUUUUUCAGUAGAUUUU
751 UGCCUUUAAAAGAAUAAAUCCCACUACAAAAAGGUAAAAUAAAAAAUCUA
801 UUCACUGAACUUACUGAUGCACUCUCCAAACGUGCCCCGUACAUCGAACG
851 ACGUGACAGAGAAAAAUACGAGUAGGCAAACAAGCCAAAAGGCAAGGGUG
901 UCCUUUCUUAAGCAUCGGUUAGGUUUGCGGGCGAUCAGUAACUGAACAAU
951 GACACAAGAUCAAGAACGUAAUUUGAGAUUUUUCAAGAUGGUUUUUUUAG
1001 GUAUCUAUUAAAACUACUUUGAUGAUCAAUACGGUAUUUUUGUCGCAUUA
1051 UUUUCCAAGCGGAAGGAACCGUGUGUUCAUUUUAUGAAUCUUGGUGUUGU
1101 AUUCACAGCUACUUCUCCUAAUGCCUUCGAUGCAUUUAGAUAAUUUUUGG
1151 AAACAUU

Terminal arm (5')

Ku arm

template

Est1 arm

Terminal arm (3')

Supp. Fig. S1F: DPhyK-TLC1

1 GAGAGGAAGAUAGGUACCCUAUGAAAAUGUCAAUGGCUGUUGCGUUUGCU
51 UAAUCGUAUUUUUUUUUUUUUCAGUCCGUGUUUUUUGUACAUUCUACGUU
101 UGAGUUUUCCAUCAUGCAGGCCUCAGAAAUUUGGUAGGCACCCGAUGGUG
151 AAGAGACAGUGUCGGCUUUCGGACCGUUCUUUUCGGUCAGAGCCUGUCGC
201 UCUUCUCUCUUCCAGAGAGUUUCGUGCACGCUGGCGUCAGUGUAGACGCC
251 UGUGCGUGCUCAAUUUGUGGUUUUUUAUUGUGUUUCUACUUAUAGAUGGC
301 UAAAAUCUGAGUUUAGAAAAUGCAAACCGUAAAUUCUUCUUUCUUCUUCU
351 UGCGUUCAGUUGCUCAACCAGUGUUUUUCCGGUUAUUCCUCCGUUUCUUU
401 CUUUCUUUCGGUCCUUUGCUCGACCUGACCUCUUAAUCACCAUGGGAAGC
451 CUACCAUCACCACACCCACACACAAAUGUUACAGCUAAUUGUUUAUUAGC
501 AAAGUUUGCACGAGUUCGCUGUUUAUUUUUUUCUCGUUUUCUUAUACCUA
551 GUAUUUUUUCUGACACUGUUUAAGGUGACAGAAAAAAAGGAGUUUAAGUU
601 AGAUUUGCAAACAGACGGUGCUAAGCGCUGUCACUUUAUGUCUAUCUUAU
651 CGUUAACUCUGGAAAAAGAAAAAGGAAAAAGAACGUCAGGGAACAUGAGU
701 AUAUAUAGAAAUGGUUUAUUCUAGUUUUUUCCGUUUUUUCAGUAGAUUUU
751 UGCCUUUAAAAGAAUAAAUCCCACUACAAAAAGGUAAAAUAAAAAAUCUA
801 UUCACUGAACUUACUGAUGAAAUUUCCAAAUGUGCCCCGUACAUCGAACG
851 AUGUGACAGAGAAAAAUACGAGUAGGUAAAUAAGCCAAAAGGCAAGGGUG
901 UCCUUUCUUAAGCAUCGGUUAGGUUUGCGGGCGAUCAGUAACUGAACAAU
951 GACACAAGAUCAAGAACGUAAUUUGAGAUUUUUCAAGAUGGUUUUUUUAG
1001 GUAUCUAUUAAAACUACUUUGAUGAUCAAUACGGUAUUUUUGUCGCAUUA
1051 UUUUCCAAGCGGAAGGAACCGUGUGUUCAUUUUAUGAAUCUUGGUGUUGU
1101 AUUCACAGCUACUUCUCCUAAUGCCUUCGAUGCAUUUAGAUAAUUUUUGG
1151 AAACAUU

Terminal arm (5')

Ku arm

template

Est1 arm

Terminal arm (3')

Supplemental Figure S2

Mfold secondary structure predictions of DA-TLC1 alleles

Mfold secondary structure predictions without constraints, with *P-num* output.

(A) TLC1

(B) DA-TLC1

(C) DET-TLC1

(D) DK-TLC1

(E) DPhyK-DA-TLC1

(F) DPhyK-TLC1

Supp. Fig. *S*2A: TLC1

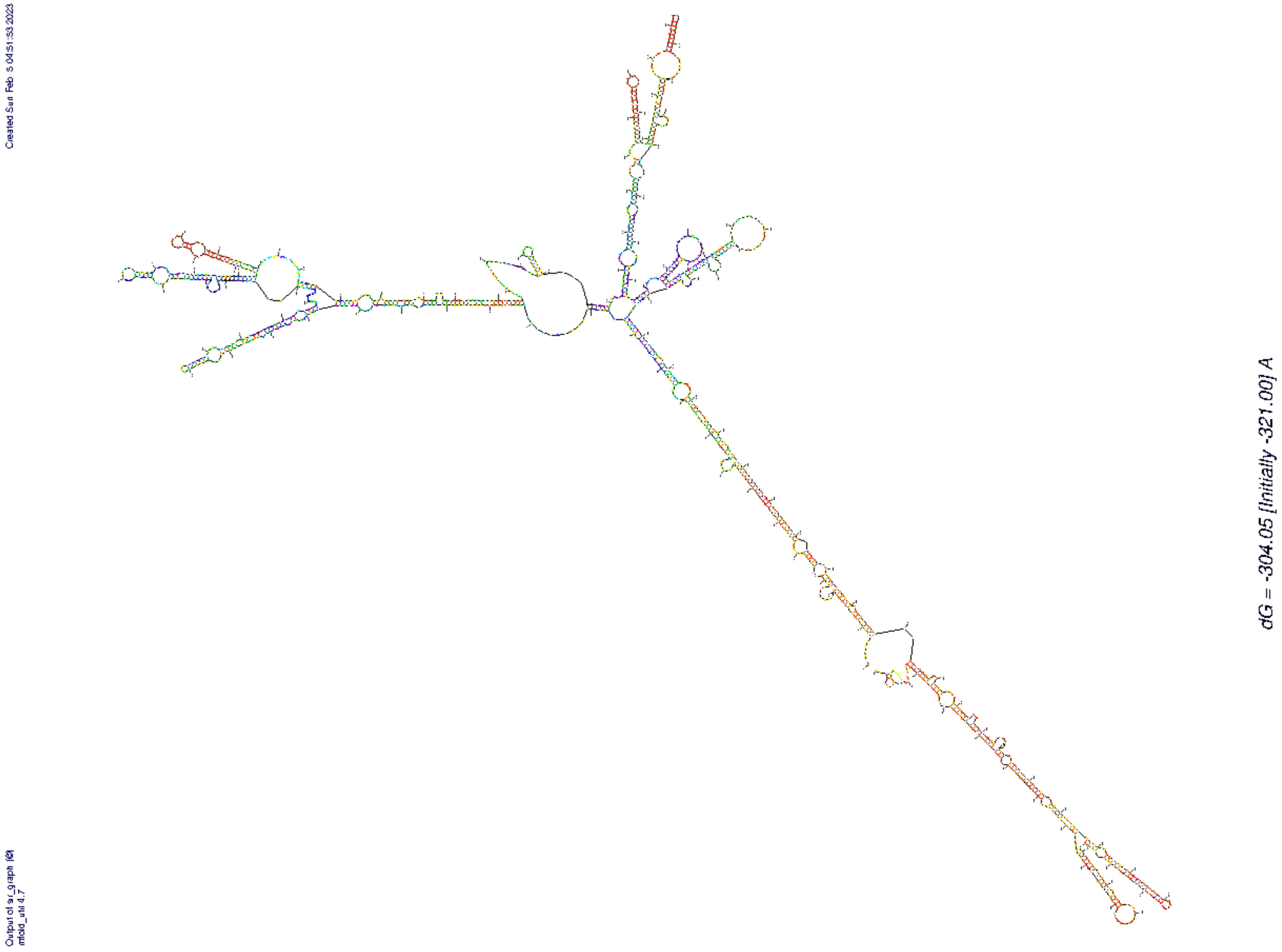

Supp. Fig. S2B: DA-TLC1

Output of sir_graph (©)
mfold_util 4.7

Created Sun Feb 5 04:52:17 2023

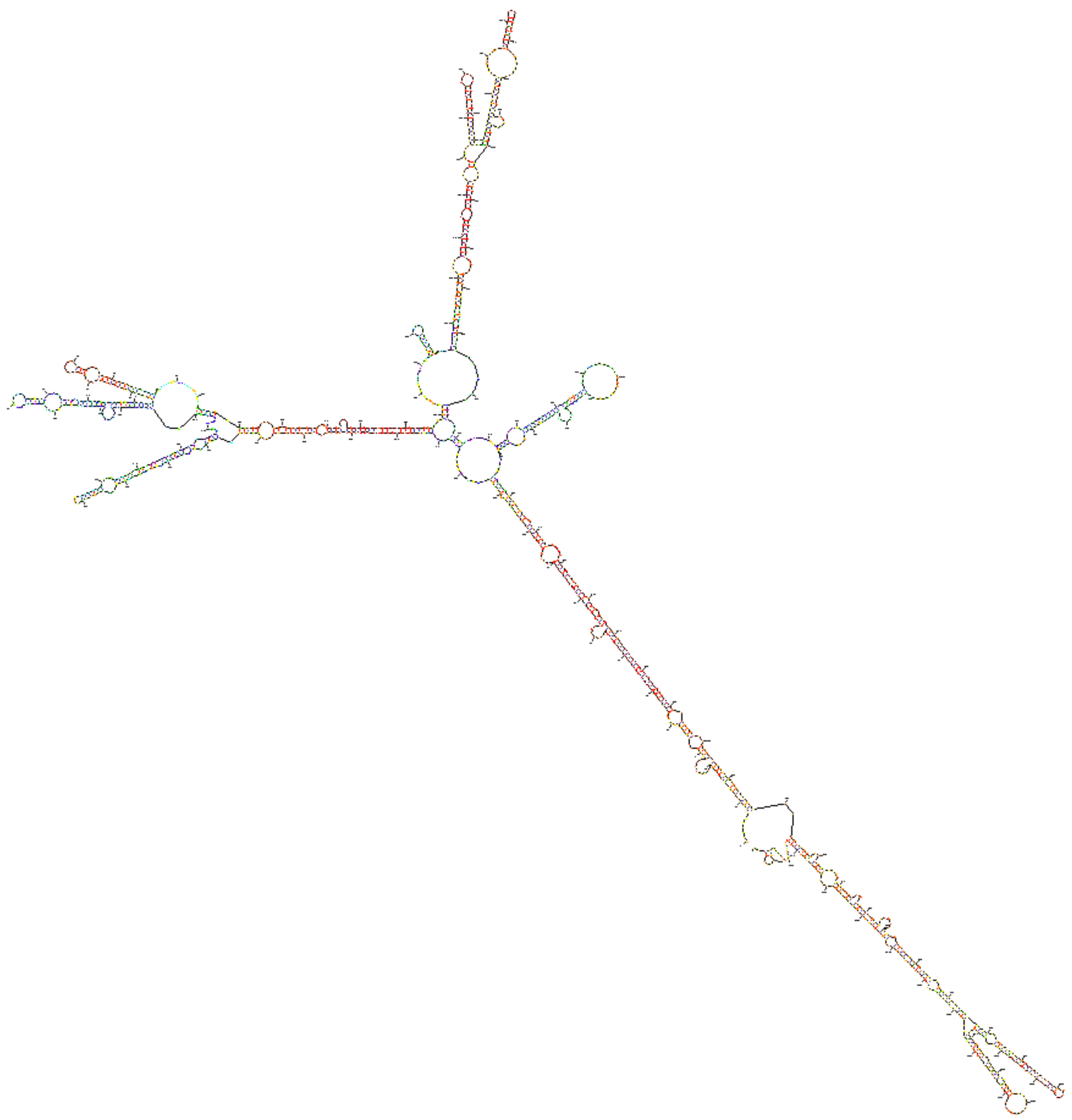

dG = -359.52 [Initially -382.20] B

Supp. Fig. S2C: DET-TLC1

Output of sir_graph (©)
mfold_util 4.7

Created Sun Feb 5 04:54:27 2023

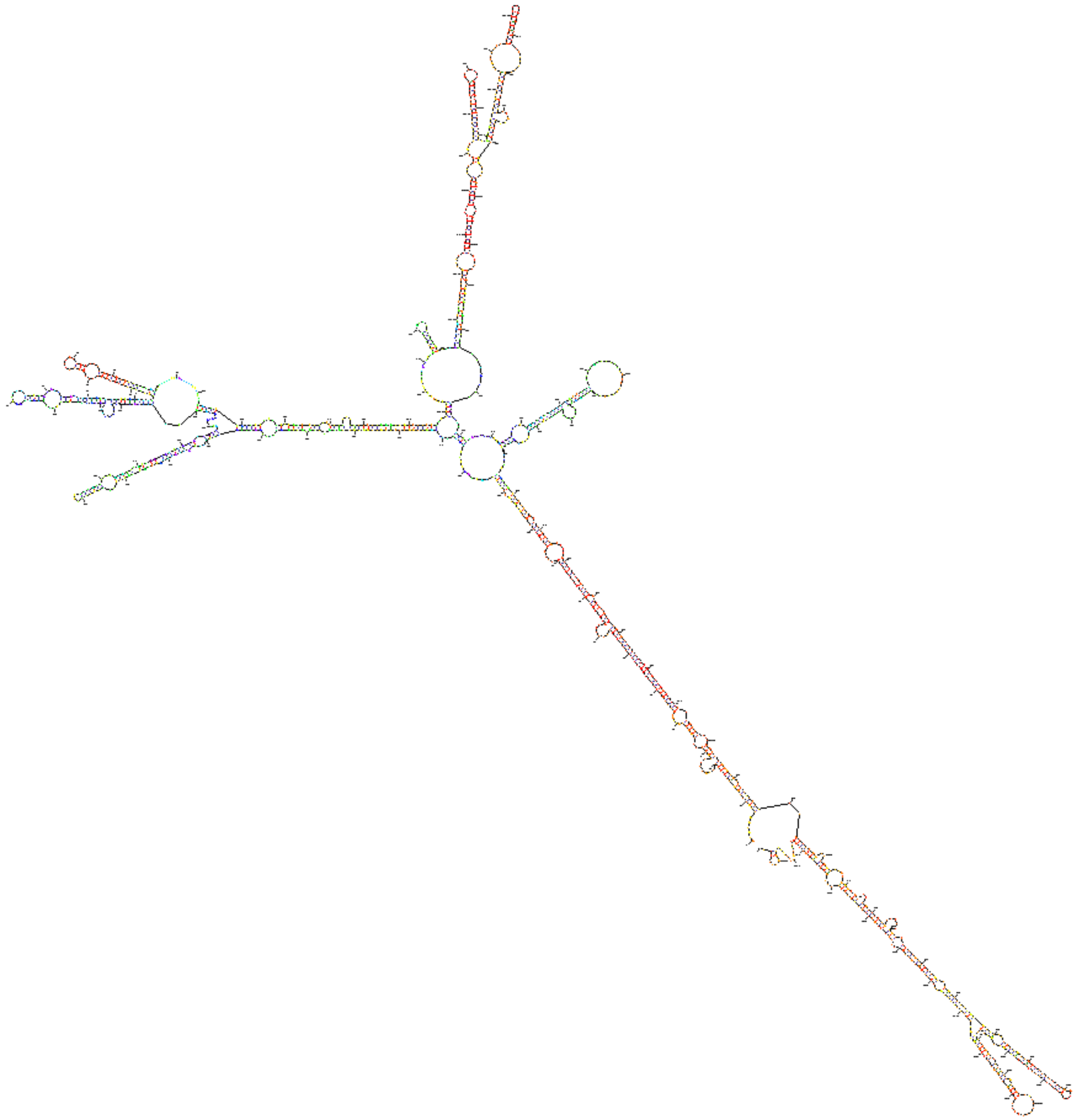

dG = -346.96 [Initially -367.30] C

Supp. Fig. S2D: DK-TLC1

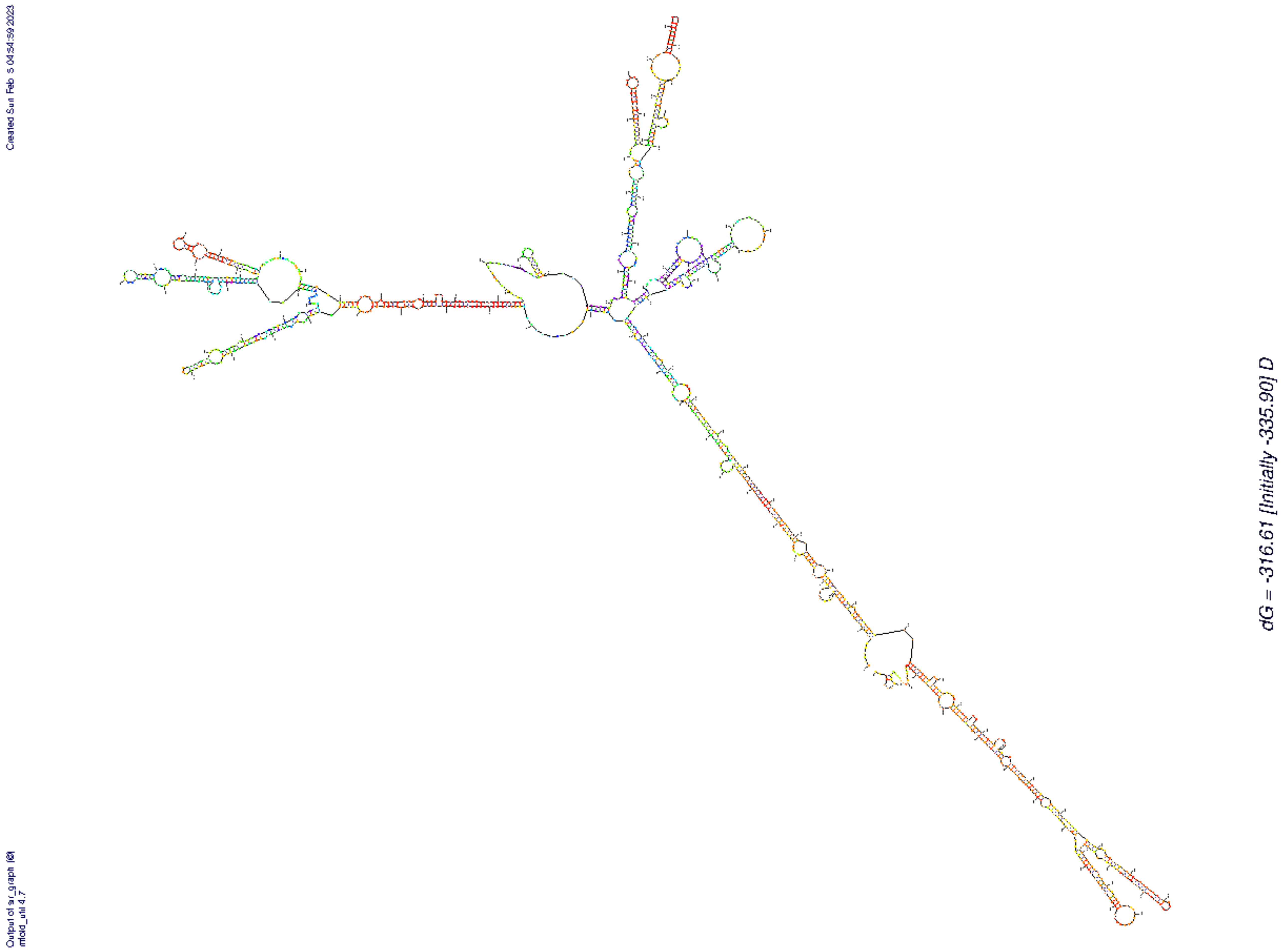

Supp. Fig. S2E: DPhyK-DA-TLC1

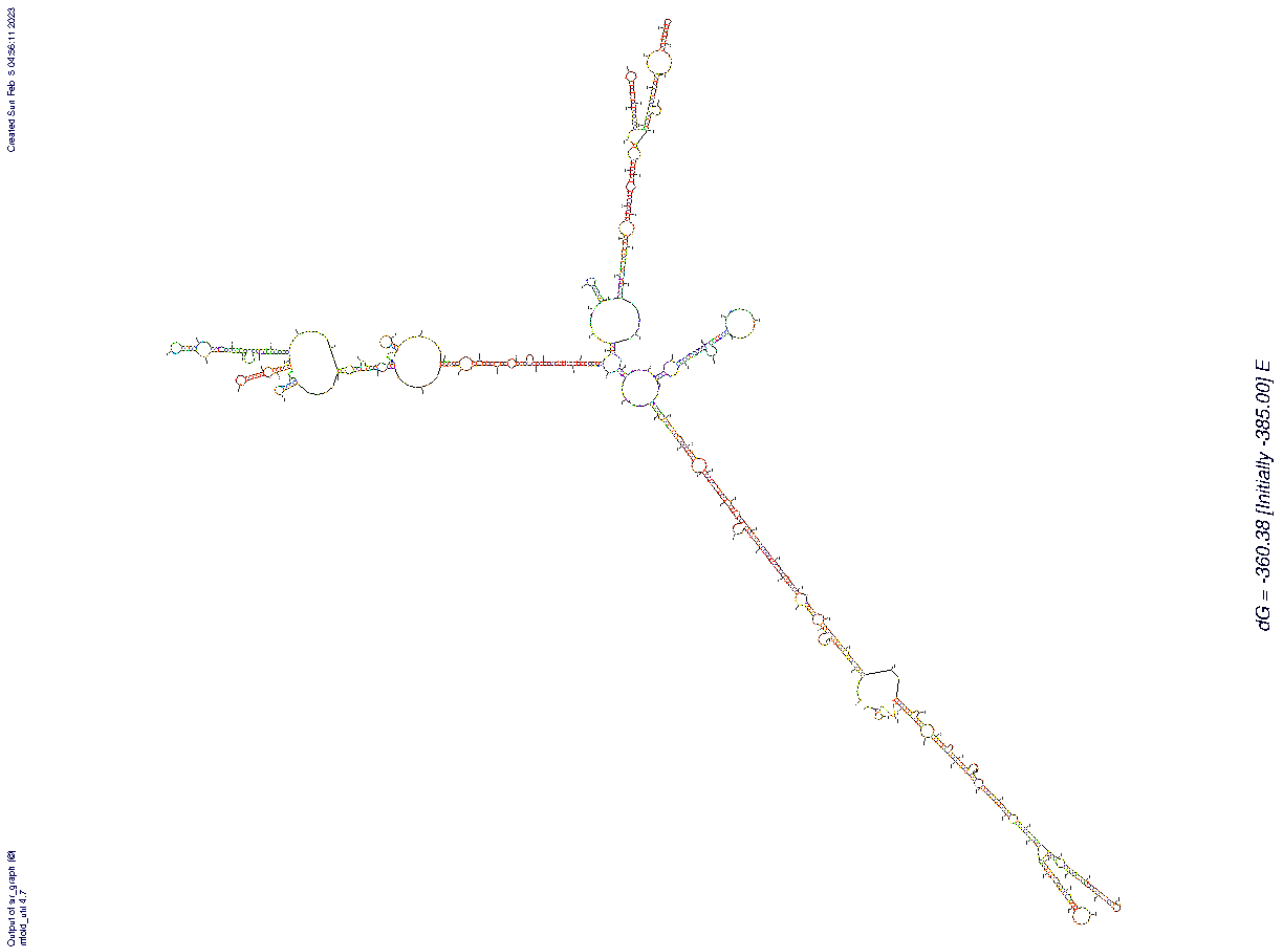

Supp. Fig. S2F: DPhyK-TLC1

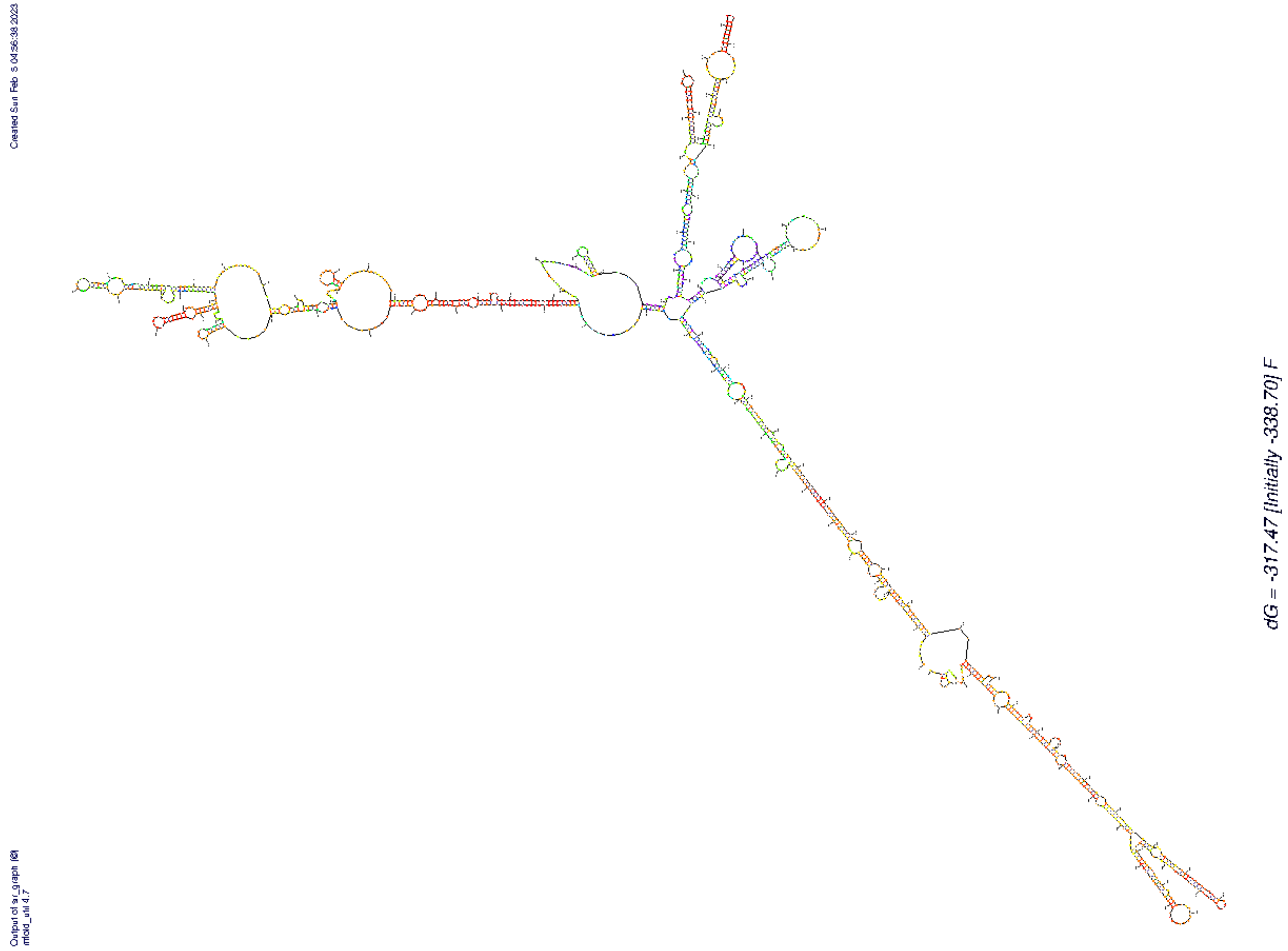

Supplemental Figure S3

Comparison of *Mfold* secondary structure predictions for wild-type, inverse-designed, and phylogenetic models of TLC1 arms. Regions of TLC1 arm secondary structures are shown, comparing the *Mfold* secondary structure prediction and phylogenetically determined (Zappulla and Cech, *PNAS* 2004) structures of wild-type TLC1 to the *Mfold*-predicted structures of the inverse-designed "determined" arms. Nucleotide changes in inverse-designed alleles are circled in red.

(A) Ku-binding arm

(B) Est1-binding arm

(C) Terminal arm

Supp. Figure S3A

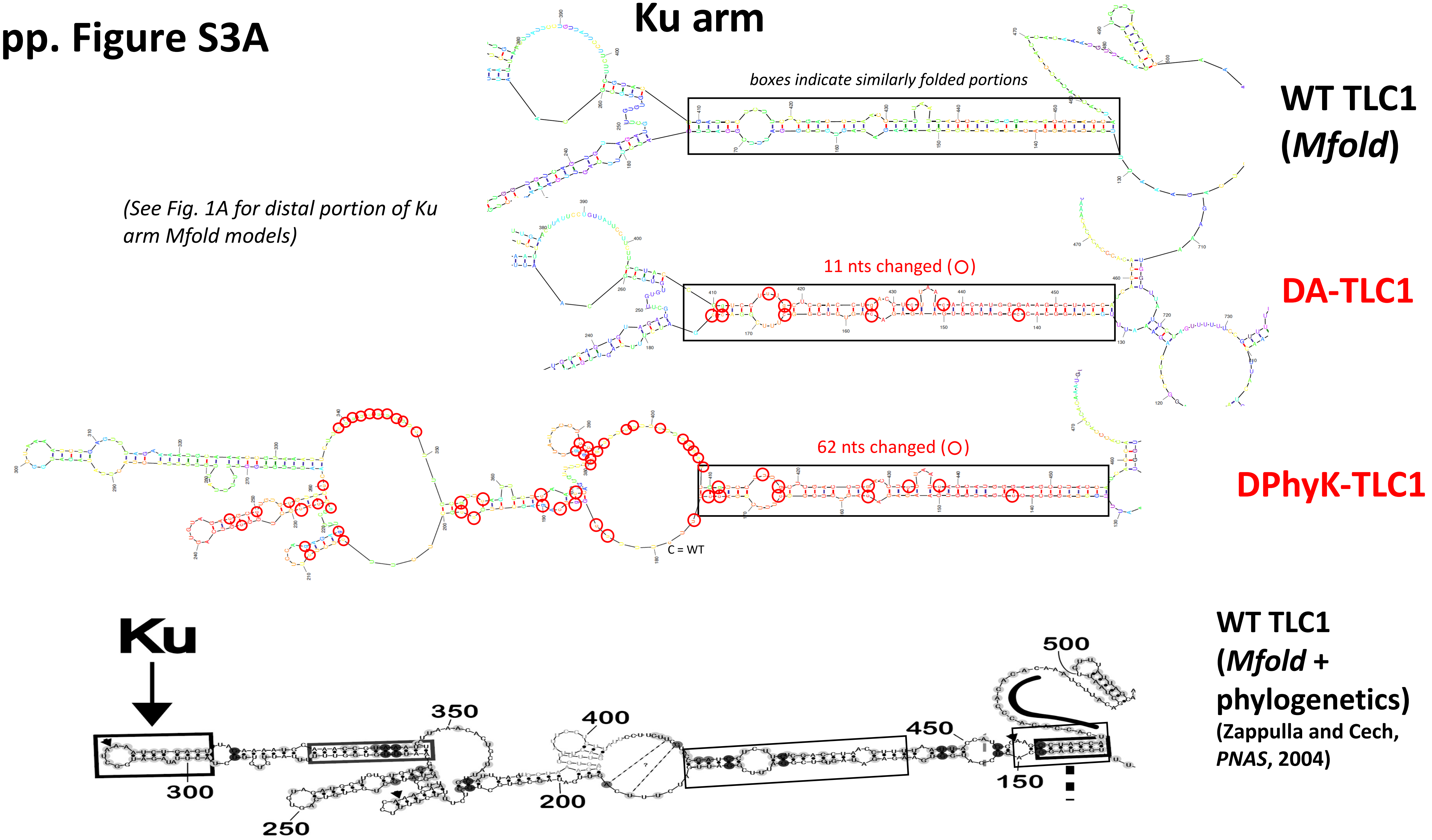

Supp. Figure S3B

(See Fig. 1A for distal portion of Est1 arm Mfold models)

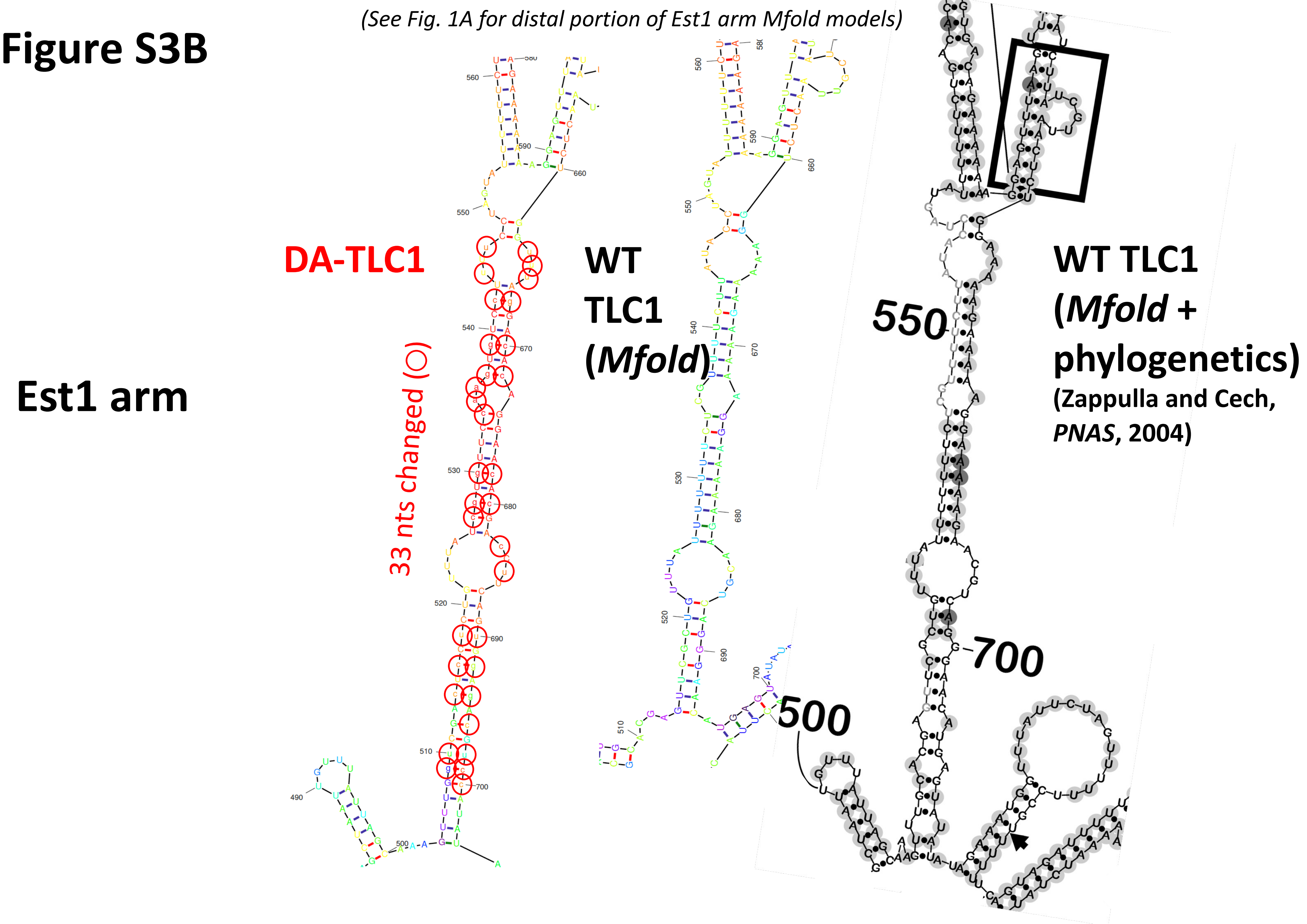

Supp. Figure S3C

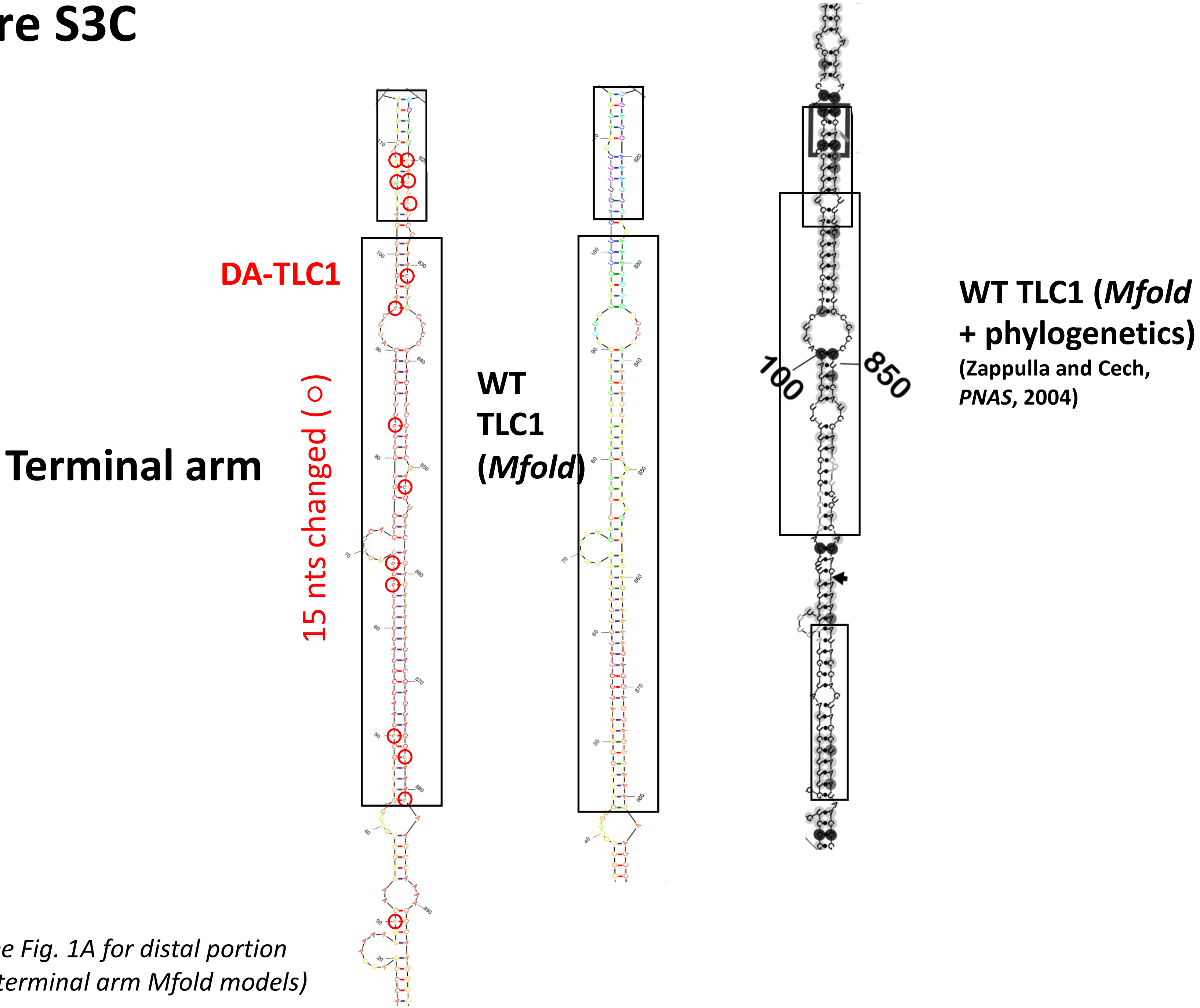

(See Fig. 1A for distal portion of terminal arm Mfold models)
